# Association of sleep behaviors, insulin resistance surrogates, and the risk of hypertension in Chinese adults with type 2 diabetes mellitus

**DOI:** 10.3389/fendo.2023.1212878

**Published:** 2023-07-20

**Authors:** Xuelin Yao, Fangting Lu, Zhen Wang, Yahu Miao, Qing Feng, Yi Zhang, Tian Jiang, Songtao Tang, Nan Zhang, Fang Dai, Honglin Hu, Qiu Zhang

**Affiliations:** Department of Endocrinology, First Affiliated Hospital of Anhui Medical University, Hefei, China

**Keywords:** type 2 diabetes mellitus, insulin resistance surrogates, hypertension, sleep duration, midday napping

## Abstract

**Objective:**

Our aim was to evaluate the association between midday napping, combined sleep quality, and insulin resistance surrogates and the risk of hypertension in patients with type 2 diabetes mellitus (T2DM).

**Methods:**

Data were collected using a standardized questionnaire. Binary logistic regression was performed to estimate the odds ratio (OR) and 95% confidence interval (CI) for the risk of hypertension. Systolic and diastolic blood pressure were grouped as categorical variables and unpaired two-sided Student’s t-test and Spearman correlation analysis were performed to estimate the association between different blood pressure levels and insulin resistance surrogates.

**Results:**

The overall prevalence rate of hypertension was 50%. Age (OR = 1.056, 95% CI:1.044–1.068), poor sleep quality (OR = 1.959, 95% CI:1.393–2.755), hyperlipidemia (OR = 1.821, 95% CI:1.462–2.369), family history of hypertension (OR = 2.811, 95% CI:2.261–3.495), and obesity (OR = 5.515, 95% CI:1.384–21.971) were significantly associated with an increased risk of hypertension. Midday napping for 1–30 min was negatively correlated with the risk of hypertension (OR = 0.534, 95% CI:0.305–0.936, P <0.05).

**Conclusion:**

Poor sleep quality and obesity are independent risk factors for hypertension. Midday napping (1–30 min) is associated with a decreased risk of hypertension in patients with T2DM.

## 1 Introduction

Type 2 diabetes mellitus (T2DM) is a serious global public health issue with high mortality and disability, affecting approximately 463 million adults living with diabetes worldwide in 2019, and is estimated to increase to 700 million by 2045 (1). T2DM has a major negative health (cardiovascular disease [CVD], diabetes-related microvascular complications, and mortality) and economic impact (2). Two-thirds of patients with T2DM have hypertension, and the incidence rate of T2DM is almost 2.5 times higher in individuals with hypertension (3). Clinically, patients with diabetes and elevated arterial blood pressure are prone to an increased incidence of microvascular and macrovascular complications (4). Therefore, T2DM prevention strategies are of paramount importance, and addressing hypertension is the key to reducing the burden of this disease.

Sleep disorders are linked to the development of hypertension, T2DM, and increased complications (5, 6). Insufficient and prolonged sleep can accelerate the onset of CVD and mortality in patients with T2DM (7). However, the association between daytime naps and hypertension in patients with diabetes remains unclear. Previous studies indicate that frequent napping is a risk factor for various negative outcomes, including cognitive decline (8), diabetes (9), and cardiometabolic diseases (10). The association between midday napping and hypertension has only recently been disputed (11, 12). For example, patients with longer naps (>60 minutes) had a considerably higher rate of hypertension in the Tongji-Dongfeng Cohort Study (13). However, Tang et al. showed that midday napping (30–60 min) was positively associated with hypertension in middle-aged and older Chinese individuals (12). Another study found that people who took longer naps (≥90 min/day) had significantly larger hazard ratios for hypertension than non-nappers (11). No report has evaluated the correlation between midday napping and hypertension in adults with diabetes. Therefore, it is important to investigate the relationship between midday napping and hypertension among patients with T2DM.

Several studies have suggested that abnormal sleep affects glucose metabolism and increases the risk of diabetes by promoting weight gain and subsequent insulin resistance (IR) (14). IR is the dysregulation of glucometabolism, which plays a critical role in the development of T2DM and hypertension (15). To evaluate individual IR levels, several studies have assessed IR surrogates, including the triglyceride-glucose (TyG) index, TyG index with body mass index (TyG-BMI), triglycerides/high-density lipoprotein cholesterol ratio (TG/HDL-c), and metabolic score for IR (METS-IR) (16, 17). Several studies have consistently revealed that higher IR surrogates are strongly correlated with the risk of hypertension in adults without diabetes (18). However, whether there are differences between different IR surrogates and the risk of hypertension in adults with diabetes remains unclear.

To evaluate the association of midday napping, nighttime sleep duration, and IR surrogates with the prevalence of hypertension among diabetic adults, we conducted a population-based study in Anhui province using the China National Diabetic Chronic Complications Study data.

## 2 Materials and methods

### 2.1 Participants and study design

Participants in this study were selected from the China National Diabetic Chronic Complications Study (China DiaChronic Study), as previously reported (19). From March 2018 to January 2020, a total of 1765 patients with diabetes who had lived in the survey area in Anhui province for at least 6 months during the 12 months enrolled in this study were recruited. All the participants provided written informed consent.

### 2.2 Inclusion and exclusion criteria

The inclusion criteria were as follows: adults aged 18 years and older who had diabetes and provided informed consent.

**Figure 1:**
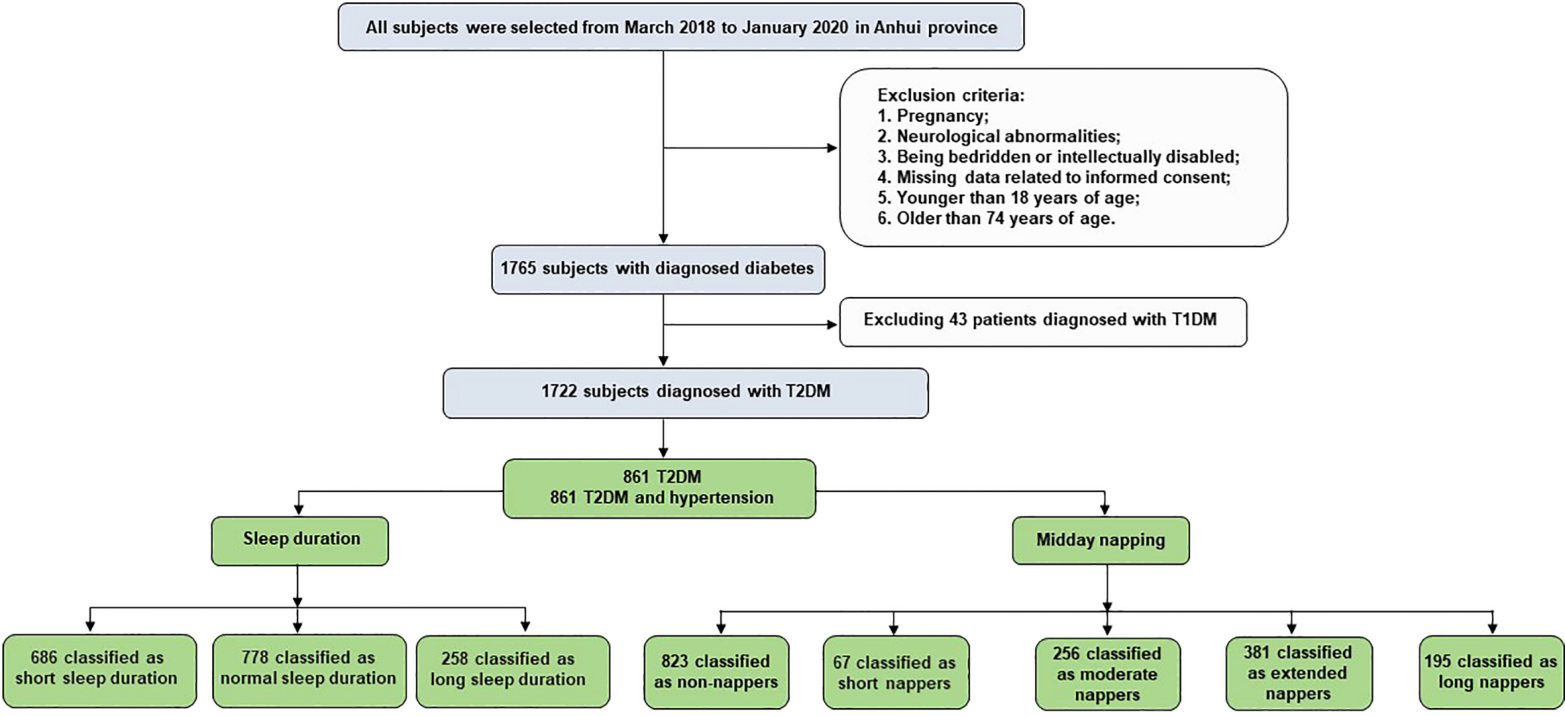
Flowchart of study participants’ selection.

The exclusion criteria were as follows: pregnancy, neurological abnormalities, being bedridden or intellectually disabled, missing data related to informed consent, and patients who were not suitable for inclusion as assessed by the study physicians (18–74 years of age). Forty-three patients with type 1 diabetes mellitus were excluded. A total of 1722 participants (861 hypertensives and 861 non-hypertensives) were included in this study for analysis. **Figure 1** presents a flowchart of the study.

### 2.3 Assessment of covariates

Age, sex, lifestyle (drinking status, smoking status, sleep duration, and sleep quality), drug administration, and personal medical history were collected at baseline by trained interviewers using semi-structured questionnaires. The body mass index (BMI) was calculated as weight in kilograms divided by height in meters squared. Systolic blood pressure (SBP) and diastolic blood pressure (DBP) were measured using an Omron blood pressure monitor. After 10 min of rest, SBP and DBP were calculated as the average of three blood pressure (BP) measurements. BMI was categorized into four groups: Underweight (BMI <18.5 kg/m^2^), Normal (18.5≤ BMI <25.0 kg/m^2^), Overweight (25.0≤ BMI <30.0 kg/m^2^), and Obese (BMI ≥30 kg/m^2^) (14). Fasting venous blood samples were collected for laboratory assays. Hyperglycemia was defined as glycosylated hemoglobin (HbA1c) >7.0% and fasting plasma glucose (FPG) >7.0 mmol/L (20). Waist circumference (WC) was calculated using a 1.5-meter soft retractable measuring tape. For Chinese adults, central obesity was defined as WC ≥90 cm in men and WC ≥85 cm in women (21). Hyperlipidemia was defined as without lipid-lowering medications, total cholesterol (TC) ≥5.20 mmol/L, triglycerides (TG) ≥1.70 mmol/L, high-density lipoprotein cholesterol (HDL-c) <1.0 mmol/L, low-density lipoprotein cholesterol (LDL) ≥3.4 mmol/L, or previously diagnosed as having hyperlipidemia by a physician (4).

### 2.4 Assessment of hypertension

Hypertension (3, 22) was defined as physician-diagnosed hypertension, or currently taking antihypertensive medications, or individuals with resting SBP over 140 mmHg and/or DBP over 90 mmHg, whereas non-hypertension was defined as individuals with SBP ≤140 mmHg and DBP ≤90 mmHg. Hypertension was further divided into three groups: Grade I (SBP 140−159 or DBP 90−99 mmHg), Grade II (SBP 160−179 or DBP 99−109 mmHg), and Grade III (SBP ≥180 or DBP ≥110 mmHg).

### 2.5 Assessment of IR surrogates

IR surrogates included the TyG index, TyG-BMI, TG/HDL-c ratio, and METS-IR. These were calculated using the following formulas: TyG index = ln [TG (mg/dL) × FPG (mg/dL)/2]; TyG-BMI = TyG index × BMI (kg/m^2^); and TG/HDL-c = TG (mg/dL)/HDL-c (mg/dL).

### 2.6 Assessment of sleep behaviors

Information on midday napping, sleep duration, and sleep quality was obtained using a self-administered questionnaire. Midday napping was assessed by asking, “How long did you take a nap after lunch on average?” According to previous studies (23), midday napping duration was categorized into five groups: non-nappers (0 min), short nappers (1–30 min), moderate nappers (30–60 min), extended nappers (60–90 min), and long nappers (≥90 min). Sleep duration was assessed using the question, “How many hours on average do you usually sleep per night?” Sleep duration was calculated from bedtime to waking time and was categorized into three groups by hours/night: Short sleep duration (<7), Normal sleep duration (7–9), and long sleep duration (≥9).

Sleep quality was assessed using the question, “Did you have the following sleep problems at least 3 days per week in the past 30 days?” with the following answer options: snoring or trouble breathing, difficulty falling asleep (taking ≥30 min to fall asleep), can fall asleep but awakening two or more times, waking up early and finding it difficult to fall asleep again, and using sleeping pills (Western medicine or traditional Chinese medicine) for at least one day in the past 30 days to help you sleep. Every answer option had the following response options: yes (poor sleep group) or no (good sleep group).

### 2.7 Statistical analysis

Continuous variables are presented as mean (standard deviation) and compared using the t-test. Categorical variables were summarized as count (%) and analyzed using the χ2 test. Statistical differences between groups for continuous variables were compared using a one-way analysis of variance (ANOVA) or unpaired two-sided Student’s t-test. ANOVA with Tukey’s test for multiple comparisons was conducted to compare the values (SBP, DBP, FPG, and HbA1c) and trends according to disease duration among the sleep groups. Spearman’s correlation coefficient was used to assess the correlations between the clinical parameters (BMI, FPG, HbA1c, HDL-c, LDL, TC, and TG), four IR surrogates (TyG, TG/HDL-c, TyG-BMI, and METS-IR), and SBP or DBP. After adjusting for control variables, binary logistic regression was used to explore the relationships among midday napping, IR surrogates, and hypertension. All analytical processes were performed using the R software (version 4.2.2), GraphPad (version 8.0), and SPSS (version 25.0). The significance threshold was set at 0.05, and all significance assessments were two-sided.

## 3 Results

### 3.1 Baseline characteristics of included participants

As shown in **Table 1**, 1,722 participants with T2DM were included in the final analysis (849 men and 873 women). The mean age of all participants was 57.16 ± 9.84 years. The prevalence of hypertension was 50% (861/1722). Among the baseline characteristics of the participants, age, WC, diabetes duration, percentage of overweight and obese individuals, proportions of coronary vascular disease, ischemic stroke, hypertension, hyperlipidemia, family history of several diseases (including cardiovascular disease, ischemic stroke, and hypertension), and percentage taking cardiovascular drugs and lipid-lowering medications were higher in hypertensive participants than in non-hypertensive participants. However, the FPG, HbA1c, and HDL-C levels were lower in the hypertensive population. In the comparison of sleep behaviors, participants with hypertension were more likely to have short sleep duration (<7 hours), longer midday napping (≥30 minutes), and poorer sleep quality. In the comparison of the four IR surrogates, the TyG-BMI and METS-IR values were higher in the hypertensive population (all P <0.05).

**Table 1:** Characteristics of the participants.

Variables (mean (SD) or N (%))	T2DM (n=861)	T2DM and Hypertension (n=861)	*P* value
**Age, years**	54.98(10.24)	59.33(8.93)	**<0.001**
**Male**	442(51.3)	407(47.3)	0.092
**WC, cm**	87.85(9.58)	91.77(9.70)	**<0.001**
**FPG, mmol/L**	9.84(3.44)	9.05(2.96)	**<0.001**
**HbA1c, %**	7.77(1.85)	7.39(1.61)	**<0.001**
**TC, mmol/L**	5.22(1.26)	5.14(1.10)	0.149
**LDL, mmol/L**	3.09(0.98)	3.04(0.95)	0.252
**HDL-c, mmol/L**	1.45(0.44)	1.41(0.39)	**0.029**
**TG, mmol/L**	2.33(3.06)	2.33(2.19)	0.979
**Diabetes duration, years**	7.58(5.29)	8.61(6.02)	**<0.001**
**TG/HDL-c**	2.04(3.81)	1.98(2.73)	0.700
**TyG index**	7.88(0.79)	7.89(0.69)	0.754
**TyG-BMI**	200.23(37.82)	211.83(37.47)	**<0.001**
**METS-IR**	47.25(9.81)	49.70(9.69)	**<0.001**
**Physical activity**	566(65.7)	567(65.9)	0.959
**Sleep duration, hours**			**0.013**
**<7**	325(37.7)	361(41.9)	
**7–9**	419(48.7)	359(41.7)	
**≥9**	117(13.6)	141(16.4)	
**Midday napping, min**			**0.027**
**0**	426(49.5)	397(46.1)	
**1–30**	44(5.1)	23(2.7)	
**30–60**	123(14.3)	133(15.4)	
**60–90**	180(20.9)	201(23.3)	
**≥90**	88(10.2)	107(12.4)	
**Sleep quality**			**<0.001**
**Poor**	713(82.8)	798(92.7)	
**Good**	148(17.2)	63(7.3)	
**BMI, kg/m2**			**<0.001**
**Underweight**	10(1.2)	3(0.3)	
**Normal weight**	403(46.8)	267(31.0)	
**Overweight**	361(41.9)	442(51.3)	
**Obese**	87(10.1)	149(17.3)	
**Current drinking**	241(28.0)	240(27.9)	0.957
**Current smoking**	173(20.1)	167(19.4)	0.716
**Family history of hypertension**	442(51.3)	625(72.6)	**<0.001**
**Family history of diabetes**	388(45.1)	382(44.4)	0.771
**Family history of obesity**	264(30.7)	291(33.8)	0.164
**Family history of cardiovascular disease**	146(17.0)	201(23.3)	**0.001**
**Family history of ischemic stroke**	177(20.6)	217(25.2)	**0.022**
**Family history of hyperlipidemia**	182(21.1)	222(25.8)	**0.023**
**Coronary vascular disease**	42(4.9)	102(11.8)	**<0.001**
**Ischemic stroke**	57(6.6)	172(20.0)	**<0.001**
**Hyperlipidemia**	500(58.1)	623(72.4)	**<0.001**
**Cardiovascular drugs**	39(4.5)	93(10.8)	**<0.001**
**Lipid lowering medication**	65(7.5)	181(21.0)	**<0.001**
**Glucose lowering medication**			0.278
**Insulin**	94(10.9)	108(12.5)	
**Oral hypoglycemic drugs**	654(76.0)	625(72.6)	
**No drug**	113(13.1)	128(14.9)	

Data are Number(%) for categorical variables, and mean (SD) for continuous variables.

P values were derived from analysis of variance or Mann-Whitney U tests for continuous variables according to data distribution and χ2 test for category variables.

WC, waist circumference; BMI, body mass index; SD, standard deviation; SBP, systolic blood pressure; DBP, diastolic blood pressure; FPG, fasting plasma glucose; HbA1c, glycosylated hemoglobin;TG, triglyceride; HDL-c, high-density lipoprotein cholesterol; LDL, low density lipoprotein cholesterol; TC, total cholesterol;TG/HDL-c, triglycerides/high-density lipoprotein cholesterol ratio; TyG index, Triglyceride-glucose index; TyG-BMI, TyG index with body mass index; METS-IR, metabolic score for insulin resistance.

P <0.05 is highlighted in bold.

As shown in **Table 2**, among the participants, 47.79%, 3.89%, 14.87%, 22.13%, and 11.32% reported a midday napping duration of 0, 1–30, 30–60, 60–90, and ≥90 min, respectively. Compared with participants reporting no napping and napping longer than 30 minutes, WC, SBP, and METS-IR, the proportion of patients with hypertension, and the percentage of participants taking antihypertensive drugs was lowest in the participants reporting midday napping of 1–30 min (all P <0.05).

**Table 2:** Characteristics of the participants stratified by midday napping.

Variables (mean (SD) or N (%))	Midday napping, min					
	0 (n=823)	1–30 (n=67)	30–60 (n=256)	60–90 (n=381)	≥90 (n=195)	*P* value
**Age, years**	56.68(9.69)	57.04(8.13)	57.97(9.40)	57.85(10.14)	56.78(10.90)	0.213
**Male**	359(43.6)	34(50.7)	132(51.6)	216(56.7)	108(55.4)	**<0.001**
**WC, cm**	89.35(9.51)	85.96(10.19)	89.03(9.75)	90.72(9.72)	92.30(10.70)	**<0.001**
**SBP, mmHg**	149.79(22.30)	143.12(18.27)	143.80(19.76)	148.09(21.05)	146.24(18.51)	**<0.001**
**DBP, mmHg**	83.83(12.06)	82.43(11.21)	82.17(12.16)	83.65(11.78)	82.62(12.07)	0.275
**FPG, mmol/L**	9.61(3.32)	9.04(2.98)	9.11(3.12)	9.39(3.20)	9.41(3.11)	0.191
**HbA1c, %**	7.62(1.70)	7.32(1.64)	7.44(1.73)	7.63(1.83)	7.62(1.78)	0.406
**TC, mmol/L**	5.20(1.16)	5.28(1.07)	5.20(1.38)	5.15(1.12)	5.07(1.18)	0.613
**LDL, mmol/L**	3.09(0.99)	3.17(0.97)	3.05(1.00)	3.02(0.93)	3.02(0.91)	0.568
**HDL-c, mmol/L**	1.45(0.42)	1.43(0.40)	1.42(0.47)	1.43(0.39)	1.37(0.41)	0.224
**TG, mmol/L**	2.26(2.03)	2.38(2.71)	2.46(3.75)	2.44(3.09)	2.23(2.31)	0.725
**TG/HDL-c**	1.88(2.47)	2.12(3.39)	2.14(3.93)	2.23(4.63)	1.90(2.21)	0.454
**TyG index**	7.92(0.70)	7.82(0.85)	7.82(0.80)	7.89(0.79)	7.85(0.73)	0.418
**TyG-BMI**	205.96(37.59)	196.66(38.83)	203.34(37.66)	208.87(37.58)	209.57(40.97)	0.096
**METS-IR**	48.15(9.63)	46.33(9.67)	48.18(9.56)	48.89(9.85)	50.18(10.79)	**0.028**
**Physical activity**	526(63.9)	42(62.7)	173(67.6)	266(69.8)	126(64.6)	0.308
**Current drinking**	213(25.9)	24(35.8)	81(31.6)	108(28.3)	55(28.2)	0.233
**Current smoking**	156(19.0)	13(19.4)	50(19.5)	76(19.9)	45(23.1)	0.788
**Antihypertensive drugs**	337(40.9)	20(29.9)	116(45.3)	175(45.9)	94(48.2)	**0.038**
**Cardiovascular drugs**	61(7.4)	2(3.0)	21(8.2)	29(7.6)	19(9.7)	0.486
**Lipid lowering medication**	119(14.5)	10(14.9)	36(14.1)	47(12.3)	34(17.4)	0.589
**Glucose lowering medication**						0.601
**Insulin**	97(11.8)	10(14.9)	32(12.5)	36(9.4)	27(13.8)	
**Oral hypoglycemic drugs**	613(74.5)	46(68.7)	194(75.8)	283(74.3)	143(73.3)	
**No drug**	113(13.7)	11(16.4)	30(11.7)	62(16.3)	25(12.8)	
**Sleep quality**						0.634
**Poor**	721(87.6)	57(85.1)	220(85.9)	337(88.5)	176(90.3)	
**Good**	102(12.4)	10(14.9)	36(14.1)	44(11.5)	19(9.7)	
**Comorbidities**						
**Coronary vascular disease**	65(7.9)	6(9.0)	21(8.2)	34(8.9)	18(9.2)	0.961
**Hypertension**	397(48.2)	23(34.3)	133(52.0)	201(52.8)	107(54.9)	**0.027**
**Ischemic stroke**	106(12.9)	8(11.9)	33(12.9)	49(12.9)	33(16.9)	0.635
**Hyperlipidemia**	551(67.0)	46(68.7)	166(64.8)	242(63.5)	118(60.5)	0.428

Data are Number (%) for categorical variables, and mean (SD) for continuous variables.

P values were derived from analysis of variance or Mann-Whitney U tests for continuous variables according to data distribution and χ2 test for category variables.

P <0.05 is highlighted in bold.

The proportions of participants with short, normal, and long sleep duration were 39.84%, 45.18%, and 14.98%, respectively. As shown in **Table 3**, the SBP, DBP, and TyG indices were the lowest in participants reporting short sleep duration. Compared to participants reporting short or long sleep duration, those reporting normal sleep duration (7–9 h) were younger, had better sleep, were less likely to take antihypertensive and cardiovascular drugs, and had comorbidities of hypertension and ischemic stroke. Patients with shorter sleep durations were more likely to be current drinkers than those who reported longer sleep duration (all P <0.05).

**Table 3:** Characteristics of the participants stratified by sleep duration.

Variables (mean (SD) or N (%))	Sleep duration, hours			
	<7 (n=686)	7–9 (n=778)	≥9 (n=258)	*P* value
**Age, years**	58.42(9.47)	55.62(10.01)	58.41(9.70)	**<0.001**
**Waist circumference, cm**	90.16(9.93)	89.58(9.89)	89.54(9.39)	0.470
**SBP, mmHg**	146.52(21.30)	147.16(20.67)	153.54(21.75)	**<0.001**
**DBP, mmHg**	82.27(11.74)	83.83(12.04)	84.78(12.29)	**0.005**
**FPG, mmol/L**	9.32(3.15)	9.47(3.29)	9.71(3.25)	0.243
**HbA1c, %**	7.52(1.75)	7.58(1.73)	7.72(1.75)	0.287
**TC, mmol/L**	5.13(1.15)	5.20(1.16)	5.25(1.33)	0.326
**LDL, mmol/L**	3.06(0.96)	3.08(0.95)	3.03(1.02)	0.767
**HDL-c, mmol/L**	1.44(0.41)	1.44(0.42)	1.40(0.41)	0.432
**TG, mmol/L**	2.22(2.55)	2.33(2.59)	2.65(3.09)	0.086
**Physical activity**	461(67.2)	492(63.2)	180(69.8)	0.097
**TG/HDL-c**	1.92(3.61)	2.00(3.12)	2.26(3.03)	0.385
**TyG index**	7.84(0.74)	7.88(0.75)	8.02(0.73)	**0.003**
**TyG-BMI**	205.13(38.14)	205.73(39.50)	209.39(33.15)	0.300
**METS-IR**	48.33(9.87)	48.39(10.15)	49.12(8.65)	0.526
**Male**	329(48.0)	406(52.2)	114(44.2)	0.055
**Current drinking**	201(29.3)	225(28.9)	55(21.3)	**0.036**
**Current smoking**	143(20.8)	158(20.3)	39(15.1)	0.124
**Antihypertensive drugs**	309(45.0)	310(39.8)	123(47.7)	**0.037**
**Cardiovascular drugs**	65(9.5)	46(5.9)	21(8.1)	**0.036**
**Lipid lowering medication**	109(15.9)	98(12.6)	39(15.1)	0.183
**Glucose lowering medication**				0.869
**Insulin**	81(11.8)	89(11.4)	32(12.4)	
**Oral hypoglycemic drugs**	503(73.3)	587(75.4)	189(73.3)	
**No drug**	102(14.9)	102(13.1)	37(14.3)	
**Sleep quality**				**<0.001**
**Poor**	632(92.1)	652(83.8)	227(88.0)	
**Good**	54(7.9)	126(16.2)	31(12.0)	
**Comorbidities**				
**Coronary vascular disease**	67(9.8)	53(6.8)	24(9.3)	0.105
**Hypertension**	361(52.6)	359(46.1)	141(54.7)	**0.013**
**Ischemic stroke**	107(15.6)	78(10.0)	44(17.1)	**0.001**
**Hyperlipidemia**	453(66.0)	503(64.7)	167(64.7)	0.844

Data are Number(%) for categorical variables, and mean (SD) for continuous variables.

P values were derived from analysis of variance or Mann-Whitney U tests for continuous variables according to data distribution and χ2 test for category variables.

P <0.05 is highlighted in bold.

### 3.2 Different patterns of clinical parameters and diabetes duration according to sleep duration and midday napping

**Figure 2:**
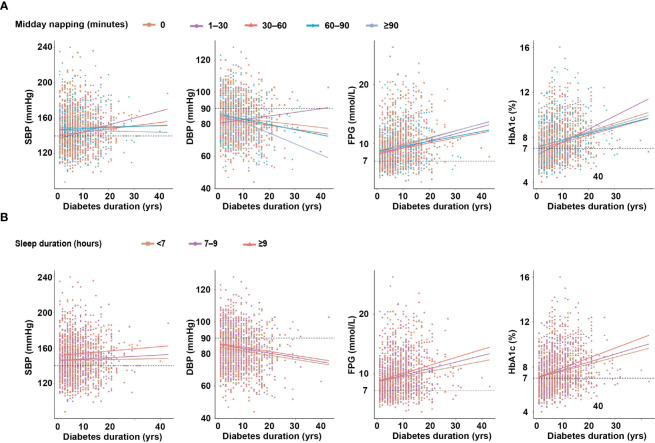
**(A)** The relationship between diabetes duration and clinical parameters according to midday napping. **(B)** The relationship between diabetes duration and clinical parameters according to sleep duration.

As shown in **Figure 2A**, the trend of increasing SBP with midday napping was comparable among the midday napping groups. DBP was inversely associated with the duration of diabetes, and the degree of decrease was more prominent in patients with midday napping ≥90 min (R^2^ = 0.094, P <0.001) than those with midday napping ≤90 minutes. FPG was positively associated with the duration of diabetes and the degree of increase was prominent in patients with midday napping ≥90 min (R^2^ = 0.048, P = 0.002) than those with midday napping ≤90 min. HbA1c was positively associated with the duration of diabetes and the degree of increase was more prominent in patients with midday napping of 1–30 min (R^2^ = 0.13, P = 0.003) than in non-nappers and those with midday napping longer than 30 min.

As shown in **Figure 2B**, the trend of increasing SBP and decreasing DBP according to diabetes duration was comparable among the sleep duration groups. FPG (R^2^ = 0.053, P <0.001) and HbA1c (R^2^ = 0.079, P <0.001) were positively associated with the duration of diabetes and the degree of increase was more prominent in patients with sleep duration ≥9 h than those with shorter sleep duration (**Table S1**).

### 3.3 Correlation between SBP or DBP and BMI, FPG, lipid parameters, and IR surrogates

**Figure 3:**
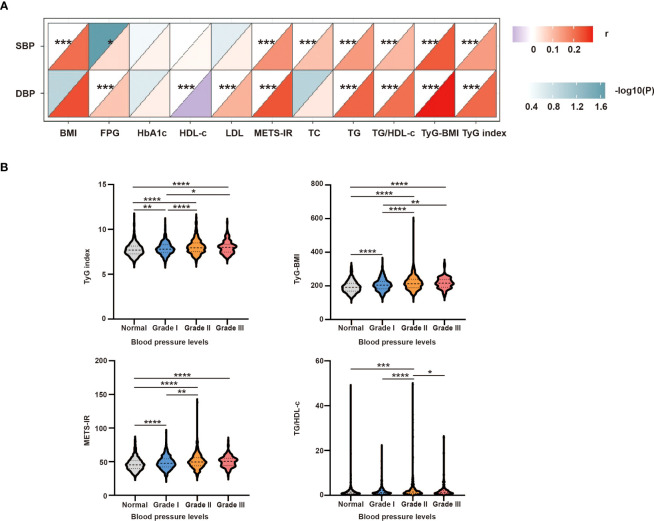
**(A)** Correlation between SBP or DBP and clinical parameters. The correlation was conducted by correlation heatmap. Both correlation coefficients are scaled such that they range from –1 to +1, where zero indicates that there is no linear or monotonic association, and the relationship becomes stronger as the coefficient approaches an absolute value of one. The upper triangle was the correlation P-value (color and significance), and the lower triangle was the correlation coefficient (r). P-values are from Spearman’s analysis. * indicates P-value <0.05. ** indicates P-value <0.01. **(B)** Intergroup differences in four insulin-resistance surrogates according to different blood pressure levels. P-values are from unpaired two-sided Student’s t-test. * indicates P-value <0.05, ** indicates P-value <0.01, *** indicates P-value <0.001, and **** indicates P-value < 0.0001.

To further analyze the correlation between SBP/DBP and the clinical parameters, a correlation heatmap was generated. As shown in **Figure 3A**, Spearman correlation analysis suggested that SBP was positively correlated with BMI (r = 0.207, P <0.0001), FPG (r = 0.055, P = 0.021), TC (r = 0.094, P <0.0001), TG (r = 0.149, P <0.0001), TgG index (r = 0.145, P <0.0001), TyG-BMI (r = 0.234, P <0.0001), TG/HDL-c (r = 0.109, P <0.0001), and METS-IR (r = 0.161, P <0.0001). Similarly, DBP was positively related to FPG (r = 0.087, P <0.0001), LDL (r = 0.114, P <0.001), TG (r = 0.221, P <0.0001), TgG index (r = 0.215, P <0.0001), TyG-BMI (r = 0.294, P <0.0001), TG/HDL-c (r = 0.203, P <0.0001), and METS-IR (r = 0.241, P <0.0001) and negatively related to HDL-c (r = -0.096, P <0.001) (**Table S2**).

### 3.4 Differences in four IR surrogates for different blood pressure levels

To further study the intergroup differences among the four IR surrogates at different blood pressure levels, the hypertensive group was further divided into grades I–III. As shown in **Figure 3B**, the TyG index, TyG-BMI, and METS-IR were higher in grade I–III group than in the normal group, and TG/HDL-c was higher in the grade II group than in the normal group. The TyG index and TyG-BMI were higher in grade II and grade III groups than in the grade I group. The TG/HDL-c and METS-IR levels were higher in the grade II group than in the grade I group. Moreover, the TG/HDL-c ratio was higher in the grade III group than in the grade II group (all P <0.05).

### 3.5 Risk factors of hypertension in participants with DM

**Figure 4:**
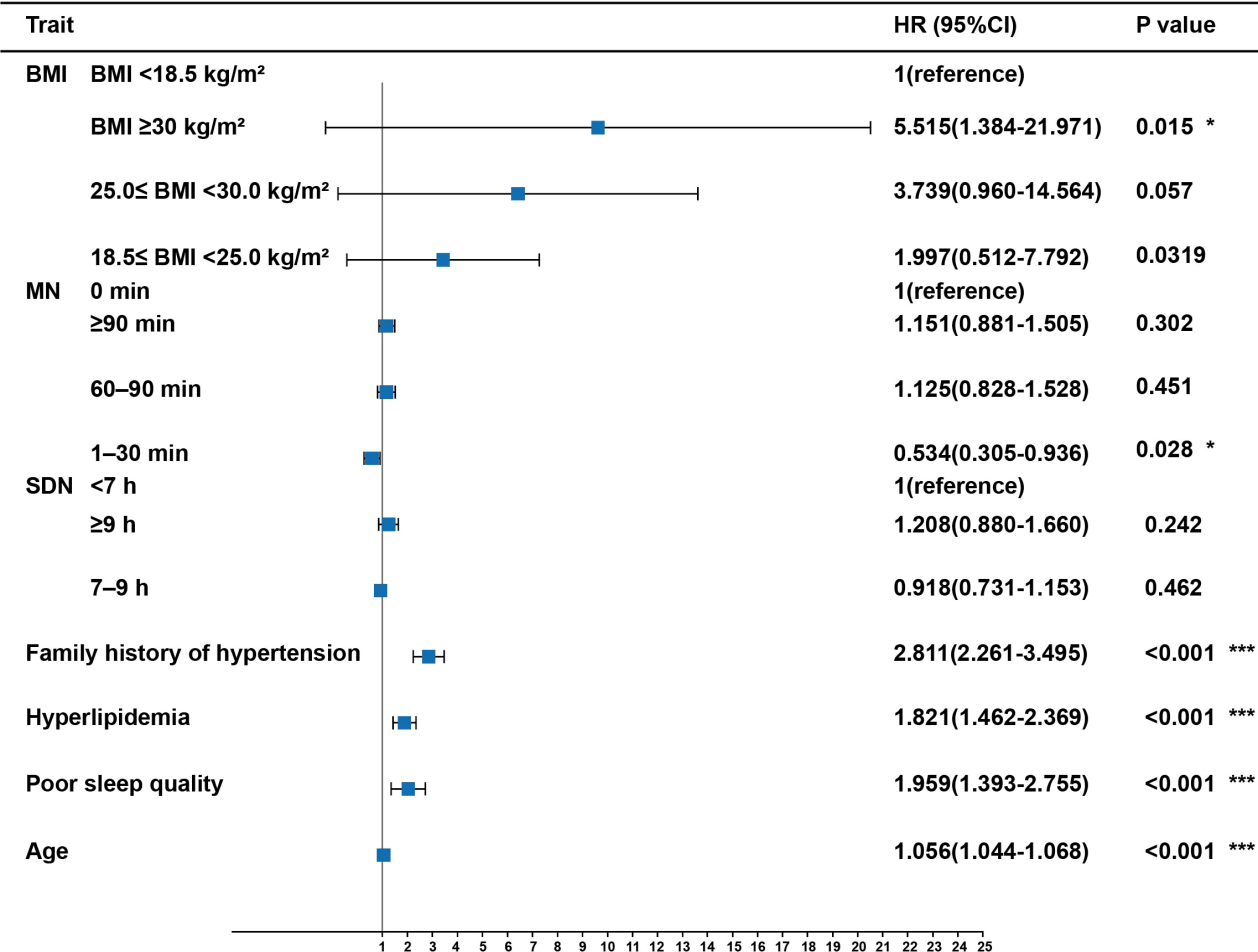
Risk factors of hypertension in participants with T2DM. Midday napping, sleep quality, sleep duration, hyperlipidemia, family history of hypertension, and BMI were taken as categorical variables, with the first as the reference group; age was taken as a continuous variable. P values are from binary logistic regression analysis. * indicates P-value <0.05. *** indicates P-value <0.001. MN, Midday napping; SDN: Sleep duration.

As shown in **Figure 4**, logistic regression analysis suggested that the risk of hypertension increased dramatically with age (OR = 1.056, 95% CI:1.044–1.068, P <0.001), poor sleep quality (OR = 1.959, 95% CI:1.393–2.755, P <0.001), hyperlipidemia (OR = 1.821, 95% CI:1.462–2.369, P <0.001), family history of hypertension (OR = 2.811, 95% CI:2.261–3.495, P <0.001), and obesity (OR = 5.515, 95% CI:1.384–21.971, P <0.05). Furthermore, the risk of hypertension decreased significantly with midday napping for 1–30 min (OR = 0.534, 95% CI:0.305–0.936, P <0.05). However, the risk of hypertension did not differ among sleep duration, normal BMI, overweight, non-napping, and napping for >30 min (P >0.05). There was no significant correlation between the four IR surrogates and the risk of hypertension (**Table S3**).

## 4 Discussions

In the present study, we report empirical results on the effects of nighttime and habitual midday nap duration on the risk of hypertension among T2DM adults. The number of participants with abnormal sleep duration was significantly higher in the hypertensive group than in the non-hypertensive group. Sleep disorders and abnormal sleep duration may contribute to IR and are associated with an increased incidence of T2DM and hypertension (9, 12–14). Insufficient sleep duration was confirmed to induce the incidence of hypertension, and the risk of hypertension was reduced by 0.3207% when sleep duration increased by one hour (24). A systematic review found a significant association between long sleep duration and mortality, the incidence of diabetes mellitus, cardiovascular disease, stroke, coronary heart disease, and obesity (25). Cohort studies have found a higher prevalence of hypertension among respondents who napped frequently and took longer daily siestas (12). Data from the UK Biobank also support the idea that napping may be a potential risk factor for hypertension (26). Overall sleep quality is also crucial for the development of hypertension. A meta-analysis showed that poor sleep quality was significantly associated with a greater likelihood of hypertension (27). Consistent with this, we identified an association between poor sleep quality and an increased risk of hypertension. Previous studies have demonstrated that impaired sleep quality can contribute to hypertension by increasing cardiovascular sympathetic tone regulation, affecting heart rate variability balance, disrupting circadian rhythmicity, and altering gut microbiota composition (28–30).

Several studies have revealed that nighttime sleep duration, midday napping duration, and bedtime are independently associated with the risk of hyperglycemia (9, 10). A systematic review of 97 cohort studies reported that daytime napping significantly increased the odds of diabetes, metabolic syndrome, cardiovascular disease, and mortality starting from 0 min/day, except for daytime napping <30 min/day (31). A recent study suggested midday naps (≥31 min) may increase the risk for IFG among early adolescents who have sufficient nighttime sleep (32). Midday napping (≥31 min) may be a novel risk factor for diabetes mellitus, and patients with diabetes are likely to constitute metabolic abnormalities and predispose individuals to hypertension, vascular stiffness, and associated CVD (7, 14, 15). Midday napping might increase the risk of hypertension among adults with diabetes via multiple plausible mechanisms such as changes in energy intake and expenditure, hormonal changes, IR, inflammation, or gut microbiota (21, 30). In our study, longer afternoon nap durations (≥30 min) were significantly increased in the hypertensive group compared to the non-hypertensive group. To some extent, the three IR surrogates (TyG index, TyG-BMI, and METS-IR) were higher in the grades I–III groups than in the non-hypertensive group. Among middle-aged and older Chinese adults, relative to non-nappers, people who had longer midday nap duration (≥90 min/day) were associated with significantly larger HR for hypertension and decreased napping duration may confer benefits for hypertension prevention (11). The underlying pathophysiological mechanisms that account for the causal association between napping and increased risk of hypertension need to be confirmed through laboratory measurements among adults with T2DM in the future.

Interestingly, short napping (1–30 min) had a beneficial effect on hypertension in T2DM compared with non-nappers. Daytime sleepiness is considered part of the biological circadian rhythm, and napping is a countermeasure that people may take to counteract the lowest point of the afternoon circadian nadir. Daytime napping has been reported as beneficial for napping on acute cognitive performance, mood, and alertness (33, 34). Midday naps also offer a variety of benefits: memory consolidation (35), preparation for subsequent learning (36), executive functioning enhancement (37), and a boost in emotional stability (38). More specifically, a 20-min nap has been shown to be long enough to lead to benefits (39). In our study, compared to non-nappers and longer nappers, short nappers had significantly lower WC, SBP, METS-IR, and rates of hypertension. Longer nap takers (≥ 90 min) exhibited a 48% increased risk of metabolic syndrome incidence as well as 30% lower metabolic syndrome reversion, compared with individuals who reported napping for 1 to < 30 min (40).

Our study had some limitations that should be considered. First, it was cross-sectional; therefore, no causal inferences could be drawn. The population chosen for this study may not be representative of the entire Chinese population, as the participants were purposively sampled from one province of China. We demonstrated the association between hypertension and midday napping but failed to infer the causal and temporal associations between elevated blood pressure and daytime napping. Second, a methodological limitation is that our data were collected from self-administered questionnaires, which may have resulted in measurement bias. Although the questions used were widely based on validated scales, several studies consistently found poor agreement between self-reported and actigraphic sleep duration (41). Overall, actigraphy and other objective measures of napping duration are required to support our findings. Third, the psychological status and living environment of the participants and the examination for apnea syndrome were not collected or assessed during the baseline interview. Previous studies have suggested that obstructive sleep apnea syndrome and prolonged exposure to environmental noise can increase stress hormone levels, which in turn favors the development of cognitive impairment, sleep disturbances, and cerebrovascular disease (42, 43). This study has several strengths. We used data from a large well-designed cohort with elaborate measurements. Second, this is the first study to evaluate the relationship among midday napping, sleep quality, sleep duration, and hypertension in a large diabetic cohort. Moreover, this study stressed the importance of short daytime napping for the prevention of hypertension in patients with diabetes.

## 5 Conclusions

In the present study, we found that midday napping for 1-30 min was independently associated with a decreased risk of hypertension in patients with T2DM. Further prospective studies are necessary to elucidate the causal link between sleep and hypertension and identify the potential mechanisms of this relationship.

## Data availability statement

The raw data supporting the conclusions of this article will be made available by the authors, without undue reservation.

## Ethics statement

This study was approved by the Ethical Review Committee (Approval No: 2018-010) and was registered in the Chinese Clinical Trial Registry (ChiCTR1800014432). The patients/participants provided their written informed consent to participate in this study.

## Author contributions

QZ and HH designed and proposed the topic, and final approval of the manuscript. XY drafted, analyzed and interpreted this study. FL, ZW, YM, and QF collected data in this study. YZ, TJ, ST, NZ and FD critically reviewed the study. All the authors who contributed to the manuscript gave their approval for its submission.
